# Reproductive control via eviction (but not the threat of eviction) in banded mongooses

**DOI:** 10.1098/rspb.2009.2097

**Published:** 2010-03-17

**Authors:** Michael A. Cant, Sarah J. Hodge, Matthew B. V. Bell, Jason S. Gilchrist, Hazel J. Nichols

**Affiliations:** 1 Centre for Ecology and Conservation, University of Exeter in Cornwall, Penryn, Cornwall TR10 9EZ, UK; 2 Department of Zoology, University of Cambridge, Downing Street, Cambridge CB2 3EJ, UK; 3 School of Life Sciences, Napier University, Edinburgh EH10 5DT, UK

**Keywords:** reproductive conflict, skew, cooperation, punishment, eviction

## Abstract

Considerable research has focused on understanding variation in reproductive skew in cooperative animal societies, but the pace of theoretical development has far outstripped empirical testing of the models. One major class of model suggests that dominant individuals can use the threat of eviction to deter subordinate reproduction (the ‘restraint’ model), but this idea remains untested. Here, we use long-term behavioural and genetic data to test the assumptions of the restraint model in banded mongooses (*Mungos mungo*), a species in which subordinates breed regularly and evictions are common. We found that dominant females suffer reproductive costs when subordinates breed, and respond to these costs by evicting breeding subordinates from the group *en masse*, in agreement with the assumptions of the model. We found no evidence, however, that subordinate females exercise reproductive restraint to avoid being evicted in the first place. This means that the pattern of reproduction is not the result of a reproductive ‘transaction’ to avert the threat of eviction. We present a simple game theoretical analysis that suggests that eviction threats may often be ineffective to induce pre-emptive restraint among multiple subordinates and predicts that threats of eviction (or departure) will be much more effective in dyadic relationships and linear hierarchies. Transactional models may be more applicable to these systems. Greater focus on testing the assumptions rather than predictions of skew models can lead to a better understanding of how animals control each other's reproduction, and the extent to which behaviour is shaped by overt acts versus hidden threats.

## 1. Introduction

An important goal of research on social evolution is to determine how conflict over reproduction is resolved in cooperatively breeding groups, and what strategies animals can employ to control each others' behaviour (Frank 2003; Ratnieks *et al*. 2006; Reeve & Shen 2006; Buston & Zink 2009; Cant & Johnstone 2009; Clutton-Brock 2009). In some species, dominant individuals suppress subordinate reproduction by interfering with mating, monopolizing access to breeding resources, inducing physiological stress or killing subordinate offspring (Digby 2000; Vehrencamp 2000; Magrath *et al*. 2004; Saltzman *et al*. 2008; Young *et al*. 2008; Hodge 2009; Young 2009). Where these direct forms of control are inefficient or unfeasible, current theory suggests that dominants will use the threat of eviction to place an upper limit on the level of subordinate reproduction (the ‘restraint’ model; Johnstone & Cant 1999; Johnstone 2000; Cant 2006; Buston *et al*. 2007). The power of eviction threats to influence behaviour has been convincingly demonstrated by recent studies of fish size hierarchies in which subordinates restrain their growth to avoid eviction by their immediate dominant (Ang & Manica in press; Buston 2003; Buston & Cant 2006; Wong *et al*. 2007, 2008). In cooperative vertebrates and primitively eusocial insects, dominant individuals often evict subordinates from groups (Johnstone & Cant 1999), but it remains unknown whether the threat of eviction can be used to induce reproductive restraint in the manner assumed by the models.

Banded mongooses (*Mungos mungo*) are an ideal species to address this shortfall in knowledge because subordinates breed regularly and evictions are common. The species lives in stable, mixed-sex groups of around eight to 40 individuals that defend territories year-round and breed two to four times per year (Cant 2000; Gilchrist *et al*. 2004). Each group contains a core cohort of between one and five dominant females (mean ± s.d. = 2.90 ± 0.91) that are older than the other females in the group and aggressively evict younger females (Cant *et al*. 2001). All females older than nine months enter oestrus around the same time (i.e. within 10 days of each other), and mate with one or more males from within the group. Most females in the group give birth in each breeding attempt, usually on the same day (Cant 2000; Gilchrist 2006), and group members cooperate to guard and provision offspring until they are three months old (Gilchrist 2004; Hodge 2005, 2007; Bell 2007; Gilchrist & Russell 2007). This extreme birth synchrony probably reduces the ability of dominants to use infanticide as a means of reproductive control because females may risk inadvertently killing their own young if they attempt to kill the offspring of other females (Cant 2000; Gilchrist 2006). Other forms of reproductive control may also be limited, since dominant females do not interfere with the mating behaviour of subordinates and do not monopolize food resources or access to natal dens (Cant 2000; De Luca & Ginsberg 2001). In this system, therefore, eviction may be the most effective way for dominants to limit reproductive competition from subordinates.

The restraint model is based on three key assumptions: (i) that dominant individuals experience reproductive competition when subordinates reproduce, (ii) that dominants respond to reproductive competition by evicting subordinate breeders, and (iii) that subordinates respond to the threat of eviction by exercising reproductive restraint. Below we test these three assumptions using long-term data from the banded mongoose system and use a simple game-theoretical model to explore the effectiveness of threats in multimember groups.

## 2. Material and methods

### (a) Study population and data collection

We studied a population totalling over 1400 individuals living in 20 study groups in and around Mweya Peninsula, Queen Elizabeth National Park, Uganda (0°12′ S, 27°54′ E), between November 1995 and April 2008. Details of habitat and climate are given elsewhere (Cant 2000). The animals were trapped regularly using live traps, anaesthetized and fitted with colour-coded plastic collars for identification (see Cant 2000 for details). Two individuals in each group were fitted with radiocollars. Most groups were habituated to walking observers (from less than 5 m). Behavioural observations were taken by two observers using hand-held Psion organizers and downloaded to the central database each evening. Most individuals were trained to step onto an electronic weighing balance in return for a small (less than 1 ml) reward of dilute milk, allowing daily weights to be collected without the need for capture. Pregnancy lasts for 60–70 days in banded mongooses and can be identified at around 40 days by swelling of the abdomen and an increase in body mass (Cant 2000; Gilchrist 2006). Birth dates can be accurately determined by a sudden change in the female's weight and body shape. For the analysis of dominant females' pup survival, dominance status was assigned on the basis of discontinuous age structure of groups and/or aggressive behaviour during eviction. In practice, dominants were readily identified by eviction behaviour and because females typically fell into two clear age cohorts, separated by a minimum of one year. Evictions were defined as cases when adult females left their group for at least one day as a consequence of aggression from older females. In 28 eviction events, aggression was either observed directly, or females were seen away from their group with conspicuous wounds. In three events, groups were not observed on the day that several females left the group, in which case eviction was assumed to have occurred on the grounds that no instances of voluntary female dispersal have been observed in our study population.

### (b) Genotyping of maternity

Maternity cannot be determined observationally, so we used microsatellite DNA analysis to determine maternity and explore the impact of breeder number on the survival of pups born to dominant mothers. DNA was extracted from tissue samples using lysis with proteinase K followed by a phenol : chloroform purification and genotyped at 14 polymorphic microsatellite loci (see electronic supplementary material for details). Maternity was assigned at 95% confidence and included all females who were known to be pregnant prior to the birth of the litter as candidate mothers.

### (c) Statistical analyses

Statistical analyses were performed in Genstat 11.1 (VSN International Ltd., Hemel Hempstead, UK). Where multifactorial analyses were required, generalized linear mixed models (GLMMs) with a binomial error structure and a logit link function were used to take into account repeated sampling within individuals, breeding attempts or groups. Analyses controlled for group identity, litter identity, group size and total rainfall post-birth as appropriate. Random terms were retained in the model unless variance components were found to be zero. All statistical tests are two-tailed.

#### (i) Analysis of reproductive competition

We investigated the influence of the number of breeding females on litter survival in the den by scoring whether or not at least one pup survived to emerge from the natal den as the binomial response term in a GLMM for 306 litters born in 19 groups. For the analysis of pup survival post-emergence, we scored the number of pups that survived to independence (three months) in 214 litters born in 17 groups as the binomial response term in a GLMM, with the number of emergent pups as the denominator. Both of these survival analyses included group size (individuals greater than six months) and rainfall (mm) (both predictors of food available to pups) as covariates. Group identity was fitted in both models as a random term to control for territory quality. To assess whether any effect of female number on survival was due to variation in litter size, we also looked at the influence of litter size on pup survival to independence. The survival of 1357 pups in 220 breeding attempts in 17 groups was fitted in a binomial GLMM, with litter size at emergence included as the main term of interest. Rainfall (mm) and group size were fitted as covariates and litter and group identity were fitted as random terms.

To investigate whether the number of breeding females influenced the survival of dominant female's pups between emergence and independence, the number of pups assigned to each dominant female was fitted as the binomial response term in a GLMM, with the number of emergent pups assigned to each mother as the denominator. This analysis was conducted on 165 emergent pups born to 45 dominant females over 65 breeding attempts in nine groups. Group, mother and litter identity were included as random terms. The influence of female number on the total number of pups that survived to independence (three months) per breeding female was determined using a non-parametric Kruskal–Wallis test followed by Mann–Whitney *U* tests (*n* = 306 litters in 19 groups). Non-parametric tests were necessary in this case as data showed severe heteroscedasticity.

#### (ii) Analysis of eviction

To investigate the probability of an eviction event occurring, we fitted whether an eviction event occurred in 226 breeding attempts in 15 groups as the binomial response term in a GLMM. The number of females of reproductive age was fitted as the main term of interest, group size and total rainfall in the previous 60 days were fitted a covariates and group identity was included as a random term. To investigate which females were targeted for eviction, we took all subordinate females of breeding age (greater than 10 months) in the group when an eviction occurred and fitted whether or not they were evicted as the binomial response term in a GLMM. Each female's pregnancy status at eviction (pregnant and non-pregnant) was included as the main term of interest. Female age (days), mean non-pregnant weight in the two months before eviction (g), group size, the number of females of breeding age and rainfall (mm) were included as covariates. Group, litter and female identity were included as random terms. This analysis used a dataset of 93 potential evictions involving 66 subordinate females in 19 breeding attempts in four groups.

#### (iii) Analysis of reproductive restraint

To investigate whether females were less likely to breed as the number of females of reproductive age (older than 10 months) increased, we conducted a GLMM which fitted whether or not each female in the group gave birth in a breeding attempt as the binomial response term. The number of females of reproductive age was included as the main term of interest and group size, rainfall in the 60 days prior to birth (mm), female age (months) and mean female weight in the two weeks around conception (g) were included as covariates. Group identity was included as a random term. The analysis was conducted on 258 potential birth opportunities involving 55 females in 52 litters in six groups. To assess the factors that influenced which females return from eviction, we took all females who were pregnant when evicted and fitted whether on not they returned in a binomial GLMM. This analysis used a dataset of 52 females over 15 eviction events in six groups. Pregnancy status in the week after eviction, female age (less than 2 and greater than 2 years), group size, the number of females of breeding age in the week post-eviction and rainfall in the 60 days prior to eviction (mm) were included as covariates. Group and eviction event were included as random terms.

## 3. Results

### (a) Do dominant females experience reproductive competition when subordinates breed?

**Figure 1.**
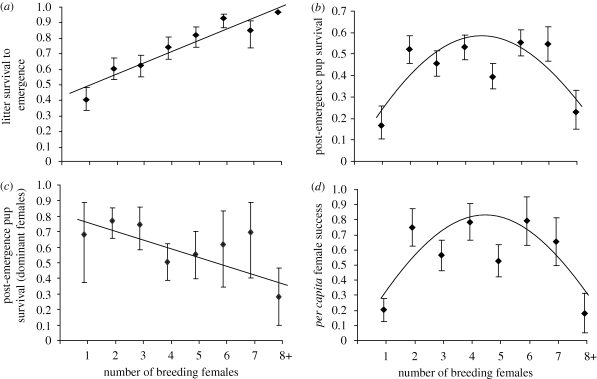
The influence of the number of breeding females on (*a*) litter survival to emergence (*n* = 306 litters from 19 groups); (*b*) the proportion of emergent pups per litter that survived to independence at age three months (*n* = 214 breeding attempts from 17 groups); (*c*) the proportion of emergent pups assigned to each dominant female (using microsatellite DNA analysis) that survived to three months (*n* = 65 breeding attempts from nine groups); and (*d*) *per capita* female success per breeding attempt (number of pups per female that survived to independence at three months; *n* = 306 breeding attempts from 19 groups). (*a*)–(*c*) show predicted means (±s.e.) and fitted model from a GLMM controlling for repeated measures among groups and (*d*) shows a quadratic regression fitted to the means.

Across all breeding attempts (*n* = 306), the average number of breeding females (±s.d.) per breeding attempt was 3.4 ± 2.0, representing a mean proportion of 0.71 (range 0.1–1.0) of adult females in each group. We tested the effect of the number of breeding females on offspring survival in two distinct phases of offspring care (Hodge 2007): first, the period for which pups remain underground in the den (up to 30 days), and second, the period between emergence and nutritional independence (90 days). Litter survival in the den (measured as the proportion of litters from which at least one pup survived) increased significantly with the number of females that bred (GLMM: 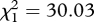, *p* < 0.001; figure 1*a*). There was no evidence, therefore, that additional breeders had a negative impact on offspring survival in the period for which offspring are kept underground.

By contrast, in the post-emergence period, pup survival initially increased and then declined when the number of breeders grew large (GLMM: number of breeding females: 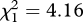, *p* = 0.043; (number of breeding females)^2^: 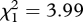, *p* = 0.047; figure 1*b*). This decline appears to reflect increased offspring competition for food in larger litters. Litter size at emergence was positively correlated with the number of breeders (linear regression: *F*_1,214_=50.19, *p* < 0.001, *R*^2^ = 18.8), and litter size had a significant influence on pup survival. Pup survival initially increased with emergent litter size, but then declined in very large litters (GLMM: emergent litter size: 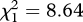, *p* = 0.004; (emergent litter size)^2^: 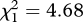, *p* = 0.032). There was evidence, therefore, that pup survival after emergence declined when many females reproduced.

The above results are based on average pup survival for all breeding females because we cannot assign mothers on the basis of behavioural observations in this species (Cant 2000; Gilchrist 2006; Bell 2007; Hodge *et al.* 2009). However, for a subset of litters, we were able to assign maternity using microsatellite DNA analysis to test directly whether dominant females experience reproductive costs when large numbers of other females breed. The proportion of pups assigned to dominant females that survived between emergence and independence declined significantly as the number of breeding females increased (GLMM: 4.52, *p*=0.036; figure 1*c*). In the post-emergence period, therefore, both mean offspring survival and the survival of dominant females' offspring specifically were lower when large numbers of females reproduced. Overall, the average *per capita* reproductive success of breeding females measured across both periods of offspring care was highest when an intermediate number of females reproduced (Kruskal–Wallis test: *H* = 34.63, *p* < 0.001; Mann–Whitney *U* tests, one versus two to seven females: *n* = 56, 179, *W* = 2066.5, *p*<0.001; two to seven versus greater than seven females: *n* =9, 179, *W* = 354, *p* = 0.003).

### (b) Do dominant females respond to reproductive competition by evicting subordinate breeders?

**Figure 2.**
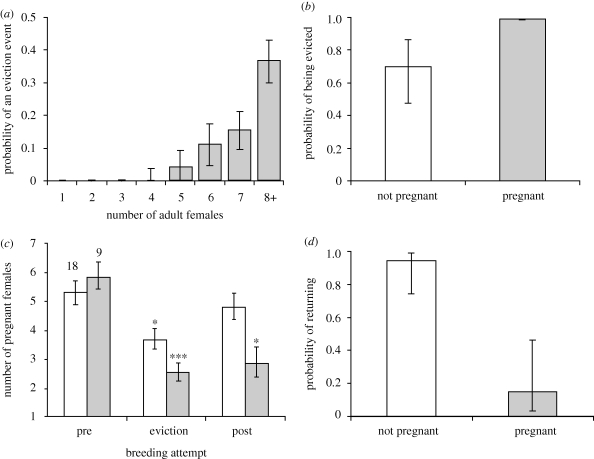
Patterns of eviction and reproduction: (*a*) probability that one or more females were evicted versus number of adult females in the group. On average six females were evicted in each event. Graph shows predicted means (±s.e.) from a GLMM controlling for repeated measures among groups (*n* = 226 breeding attempts). (*b*) Female pregnancy status versus probability of being evicted. Graph shows predicted means (±s.e.) from a GLMM controlling for repeated measures among litters, groups and individuals (*n* = 66 females). (*c*) Number of breeding females per group in the breeding attempt preceding an eviction event (‘pre’), the breeding attempt in which an eviction event occurred mid-way through gestation (‘eviction’) and the subsequent breeding attempt (‘post’). Open bars are eviction events in which all evicted females eventually rejoined the group (*n* = 18) (temporary evictions) and shaded bars are eviction events that led to permanent dispersal (*n* = 9) (permanent eviction). Asterisks indicate post hoc significance level compared with ‘pre’ bar of the respective categories (*p < 0.05; ***p < 0.001). (*d*) Probability of return to the group within one week of eviction of 52 pregnant females as a function of their pregnancy status one week after eviction. Graph shows predicted means (±s.e.) from a GLMM controlling for repeated measures among litters and groups (*n* = 52 females).

We observed a total of 31 female eviction events in seven groups, resulting in the eviction of 186 females of breeding age. The average number of females in the group prior to eviction was 8.86 (±0.39) and on average six females were evicted in each event (range 1–13). All eviction events occurred during periods when reproductive conflict between females was likely to be high: 70 per cent occurred when the group were in the latter stages of pregnancy (greater than 35 days post-oestrus) and 30 per cent occurred when the group were in oestrus. The probability that an eviction event occurred increased sharply with the number of potential breeding females in the group at that time (GLMM: 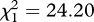, *p* < 0.001; figure 2*a*). Dominants specifically targeted reproductive subordinates for eviction: females that were pregnant were more likely to be evicted compared with those that were not (GLMM: 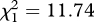, *p* = 0.006; figure 2*b*).

In nine eviction events, all evicted females permanently left the group, reducing the number of adult females in the group from 9.33 (±0.85) to 3.89 (±0.46) (paired *t*-test: *t*_8_ = 6.52, *p* < 0.001). In 18 eviction events, all evicted females eventually rejoined the group (‘temporary evictions’) and in four eviction events, some females returned and the remainder dispersed. In temporary eviction events, the number of pregnant females was significantly lower in the breeding attempt immediately following eviction than in the breeding attempt before eviction (paired *t*-test: *t*_16_ = 2.86, *p* = 0.017; figure 2*c*). For permanent evictions, the number of breeders was reduced in both of the two subsequent breeding attempts (ANOVA: *F*_2,20_ = 11.78, *p* < 0.001; figure 2*c*).

### (c) Do subordinate females respond to the threat of eviction by exercising reproductive restraint?

After controlling for a positive influence of female weight (GLMM: 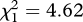, *p* = 0.034), females were not less likely to breed in a given breeding attempt as the number of females of reproductive age in the group increased (GLMM: 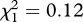, *p* = 0.73). There was no evidence, therefore, that subordinate females exercised reproductive restraint when the probability of an eviction event was high. However, pregnant subordinates that were evicted often aborted their litter within a few days and were reaccepted into the group. Of 32 females that were pregnant when evicted and later rejoined the group, 25 (78%) aborted before they were readmitted. Females that aborted their litter in the week after eviction were more likely to return than those that did not (GLMM: 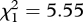, *p* = 0.025; figure 2*d*). Of those females that were evicted and readmitted, we found no difference in the probability of being visibly pregnant in the breeding attempt following eviction compared with the breeding attempt prior to eviction (proportion of evicted females breeding pre-eviction = 2.80 ± 0.60, proportion of evicted females breeding post-eviction = 3.19 ± 0.53; paired *t*-test: *t*_15_ = −0.59, *p* = 0.57). Hence, females did not respond to temporary eviction by foregoing reproduction in the subsequent breeding attempt.

## 4. Discussion

We found strong support for the first two assumptions of the restraint model, namely that subordinate reproduction is costly to dominants and that dominant individuals respond to increased reproductive competition by evicting subordinate breeders. By contrast, we found no evidence that subordinates exercise reproductive restraint to avoid being evicted in the first place. There are at least two likely reasons for the failure of eviction threats to induce pre-emptive restraint in this system. First, pregnant subordinates who were evicted could usually regain access to the group if they aborted their litter. Spontaneous abortion may be a side-effect of the physiological stress of eviction, or the proximate mechanism by which subordinates maximize their probability of readmittance (Young *et al*. 2006). In either case, the capacity of subordinates to respond retrospectively and return to the group is likely to erode any incentive to exercise reproductive restraint pre-emptively. Second, even non-pregnant subordinates were frequently evicted (figure 2*b*). This may be because dominants cannot discriminate pregnant and non-pregnant subordinates, or because it does not pay them to discriminate since females that are currently non-pregnant are nevertheless very likely to breed in the next breeding attempt. As a consequence, however, subordinate females have little to gain from cooperating by exercising pre-emptive restraint.

**Figure 3.**
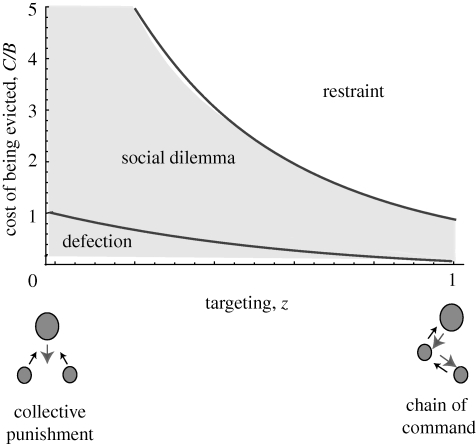
A model of reproductive restraint with two subordinates. Groups consist of a single dominant and two subordinates of equal rank. Zones for which the evolutionarily stable outcome is mutual reproductive restraint (unshaded) or defection and consequent eviction of one of the subordinates (shaded) are plotted as a function of the cost of being evicted and the degree to which dominants single out transgressors for punishment (targeting, *z*). The zones marked ‘restraint’ and ‘defection’ indicate areas where the strategies of restraint and defection are, respectively, strictly dominant, i.e. they yield the highest pay-off irrespective of the strategy of the other player. In the ‘social dilemma’ zone, defection is the Nash equilibrium solution even though both subordinates would do better if they could agree to exercise restraint. The case where *z* = 1 applies to groups that exhibit a linear hierarchical structure, such that each individual monitors and targets its immediate subordinate for punishment (labelled ‘chain of command’ in the figure). In this case, restraint is stable if the cost of being evicted outweighs the benefits of claiming additional reproduction (i.e. *C/B* > 1) (see appendix for details of the model).

This second point highlights a general issue with the use of threats to induce cooperation in multimember groups. Restraint is expected in the original two-player restraint model (Johnstone & Cant 1999) because in this case the threat of eviction is perfectly targeted: a subordinate is certain to be evicted if it claims more than the maximum tolerated by the dominant, and certain to avoid eviction if it exercises restraint. Where non-transgressors are sometimes evicted, however, the evolution of pre-emptive restraint faces a coordination problem similar to the prisoner's dilemma. This coordination problem is illustrated in figure 3, which shows the result of a simple model of reproductive restraint when groups contain two subordinates instead of one, and where dominants vary in the degree to which they single out transgressors for eviction (see appendix). In the unshaded region marked ‘restraint’, restraint by both subordinates is evolutionarily stable and no eviction occurs. In the shaded region marked ‘defection’, both subordinates do best to defect, regardless of the strategy of the other. In between there is a region marked ‘social dilemma’ where the Nash equilibrium outcome is defection (and consequent eviction), even though both subordinates would do better if they could agree to exercise restraint. The model predicts, therefore, that reproductive restraint rapidly becomes evolutionarily unstable where punishments are targeted with less than perfect accuracy (a similar conclusion is reached by Boyd & Richerson (1992) who refer to targeted punishment as ‘retribution’ to distinguish it from punishment that falls upon a group).

The model suggests that eviction threats will be more effective in dyadic relationships and dominance hierarchies in which transgressors are clearly distinguished from non-transgressors. This result helps to explain why the threat of eviction exerts such a strong influence on growth strategies in fish size hierarchies. In these hierarchies, each individual monitors and punishes its immediate subordinate. Threats of punishment are most effective in these circumstances for two reasons. First, transgressors can be identified with certainty, avoiding the social dilemma illustrated in figure 3. Second, the rewards of cooperation flow directly back to the punisher, avoiding the ‘second-order free rider’ problem (Boyd & Richerson 1992; Boyd *et al*. 2003) which hampers the spread of punishment strategies in non-hierarchical models of cooperation (Boyd & Richerson 1992; Boyd *et al*. 2003; Bowles & Gintis 2004; Panchanathan & Boyd 2004). Dominance hierarchies are common in social vertebrates and were a likely feature of ancestral hominids prior to more recent transitions to egalitarianism (as inferred from the social structure of hunter–gatherers; Boehm 1999; Svensson 2009). Economic models that feature symmetrical players and equal sharing of public goods may therefore underestimate the extent to which cooperation in these systems has been shaped by punishment and threats.

Our study shows that banded mongooses are not in the steady equilibrium state assumed by transactional models of skew. The use of eviction as a means of reproductive control, coupled with the ineffectiveness of eviction threats to induce restraint, leads to the periodic forced dispersal of adult breeders from their natal group and fluctuations in group size. By contrast, eviction is rarely observed in fish size hierarchies, precisely because the threat of eviction is effective at inducing growth restraint in these systems (Buston 2003). Observations of frequent eviction, therefore, offer good *prima facie* evidence of the ineffectiveness of eviction threats to induce cooperation. Moreover, since the factors that increase the effectiveness of exit threats also increase group stability, species in which group members form a linear hierarchy are predicted to exhibit greater genetic differentiation between groups than non-hierarchical species, other things being equal. Understanding this link between within-group conflict and social stability is important for a more general theory of social evolution because patterns of dispersal are expected to exert a strong influence on selection for helping and harming behaviour (West *et al*. 2002; Gardner & West 2006; Johnstone & Cant 2008; Gardner 2010).

In addition to the accurate targeting of punishments, effective threats require that receivers possess (or can acquire) accurate information about the location of threat thresholds and the consequences of triggering a threat. Transactional models are thus reliant on a number of critical assumptions that remain implicit in the original models, and few studies to date have elucidated these assumptions or tested whether they hold in the social system of interest. We believe that further progress in the study of reproductive skew can be gained not by focusing on correlations between skew and various model parameters, but by testing the assumptions underlying the models and identifying what behavioural strategies animals can use to control each other's social behaviour. For example, studies to test whether threats are effective can help to rule out reproductive transactions (whether based on the idea of concessions, Vehrencamp 1983; Reeve & Ratnieks 1993; or restraint, Johnstone & Cant 1999) as an explanation for the pattern of subordinate reproduction. Conversely, evidence that animals respond to exit threats (e.g. Buston 2003; Wong *et al*. 2007, 2008; Ang & Manica in press) suggests that transactional models may apply well in some systems.

## Appendix. A restraint model with two subordinates

We assume that the dominant evicts a single subordinate if the total share of reproduction claimed by the two subordinates exceeds some threshold *p*_max_. Subordinates make a simultaneous decision to adopt one of two strategies: restraint *R*, which entails the subordinate claiming a share equal to exactly *p*_max_/2; or defection *D*, which entails the subordinate claiming a share greater than *p*_max_/2. Defection is assumed to bring a constant fitness benefit *B*, provided the defector can avoid being evicted. If a subordinate is evicted, we assume that it suffers a cost *C*. The extent to which dominants target transgressors varies from *z* = 0 (implying that the dominant evicts at random with respect to subordinate behaviour) to *z* = 1 (implying that eviction is perfectly targeted, such that subordinates who exercise restraint are never evicted). If both subordinates defect, we assume that the dominant evicts one of them at random. Pay-offs *W*_*i*__,__*j*_ of strategy *i* against *j* are as follows: *W*_*R*__,__*R*_ = 0, *W*_*R*__,__*D*_ = −*C*(1 − *F*), *W*_*D*__,__*R*_ = −*CF* + *B*(1 − *F*), *W*_*D*__,__*D*_ = (*B*/2) − (*C*/2), where *F*(=(1 + *z*)/2) is the probability that a subordinate who plays *D* is evicted when paired with an *R*-strategist. Setting *B* equal to 1 and solving for the critical value of *C* for which restraint or defection are dominant strategies yields, respectively, the following equations *C*_*R*_ = 1/*z*; *C*_*D*_ = (1 − *z*)/(1 + *z*). These equations define the upper and lower curves, respectively, in figure 3.
